# Restriction Genes for Retroviruses Influence the Risk of Multiple Sclerosis

**DOI:** 10.1371/journal.pone.0074063

**Published:** 2013-09-16

**Authors:** Bjørn A. Nexø, Bettina Hansen, Kari K. Nissen, Lisa Gundestrup, Thorkild Terkelsen, Palle Villesen, Shervin Bahrami, Thor Petersen, Finn S. Pedersen, Magdalena J. Laska

**Affiliations:** 1 Department of Biomedicine, Aarhus University, Aarhus C, Denmark; 2 Bioinformatics Research Centre, Aarhus University, Aarhus C, Denmark; 3 SKAUvaccines, Incuba Science Park, Aarhus C, Denmark; 4 Department of Neurology, Aarhus University Hospital, Aarhus C, Denmark; 5 Department of Molecular Biology and Genetics, Aarhus University, Aarhus C, Denmark; National Institute of Allergy and Infectious Diseases, United States of America

## Abstract

We recently described that the autoimmune, central nervous system disease, multiple sclerosis (MS), is genetically associated with the human endogenous retroviral locus, HERV-Fc1, in Scandinavians. A number of dominant human genes encoding factors that restrict retrovirus replication have been known for a long time. Today human restriction genes for retroviruses include amongst others *TRIMs, APOBEC3s, BST2* and *TREXs*. We have therefore looked for a role of these retroviral restriction genes in MS using genetic epidemiology. We here report that markers in two *TRIMs*, *TRIM5* and *TRIM22* and a marker in *BST2*, associated statistically with the risk of getting MS, while markers in or near *APOBEC3s* and *TREXs* showed little or no effect. This indicates that the two *TRIMs* and *BST2* influence the risk of disease and thus supports the hypothesis of a viral involvement.

## Introduction

Host restriction factors for retroviruses and the underlying genes have been known for a long time. The classical example is the *Fv-1* restriction in mice that limits the infection of mouse cells with sensitive murine leukemia viruses [1] by accelerating the unpacking of the core particles from the incoming virions [2]. Similar factors, encoded by the *TRIM* genes have been detected in human cells in the investigation of HIV-infection [3]. TRIMs in general react with the capsid lattice of viruses, presumably degrading the incoming core in an ubiquitin-proteasome pathway [4]. The specificity of TRIM factors is quite broad, and many retroviruses are affected to various extents [5]. Even an interaction between TRIM22 and influenza virus was described recently [6].

Another set of restriction factors are encoded by the *APOBEC3* genes, of which there are 7 in humans, *APOBEC3A* to *APOBEC3H* [7]. There is no *APOBEC3E. APOBEC3* genes encode cytosine deaminases that are co-packaged into viral particles during formation and mutate the sequence of the viral genome [8], thus making the viral particle unable to complete a full round of infection. Again, *APOBEC3* genes have a fairly broad specificity and affect many retroviruses [7].

Yet another restriction of retroviral replication is caused by the gene *BST2*. The product of this gene, Tetherin, is a cell surface protein, which tethers budding virions to the membrane and prevents the scattering of viral particles in cell-free form [9]. Cell to cell transmission is presumably less affected. As another function it has recently been reported that Tetherin acts as a sensor for viral infection, eliciting NF-κB dependent pro-inflammatory gene expression, similarly to TRIM5α [10]. Tetherin affects different viruses, but the effect on retroviruses is variable, probably reflecting that different viruses have developed different countermeasures [9–11].

Finally, the *TREX* genes, of which there are 2 in humans, *TREX1* and *TREX2*, encode 3-prime repair exonucleases that interfere with viral DNA formation and integration [12]. Single-stranded DNA derived from endogenous retroelements accumulates in *TREX1*-deficient cells, and *TREX1* can metabolize DNA resulting from reverse transcription. *TREX1* has been shown to prevent cell-initiated autoimmune responses and mutations in the gene are underlying the defect in Aicardi-Goutieres syndrome and chilblain lupus [13].

We recently described that the autoimmune, central nervous system disease, multiple sclerosis (MS) is genetically associated with the human endogenous retroviral locus, HERV-Fc1 [14,15] in Scandinavians. Also, HERV-Fc1 extracellular RNA seems increased in plasma of MS patients. Remarkably, HERV-Fc1 was mainly increased in plasma of patients with recent histories of attacks [16].

We originally described that the marker rs3802981 in the *TRIM5* gene was associated with MS [14]. Now, we have continued this line of inquiry and report that further markers in both *TRIM5* and *TRIM22* associate with this disease. The associations were strong enough to withstand Bonferroni correction for the multiplicity of testing. Markers in *APOBEC3* also showed signs of being associated with MS, but the p-value was not low enough to withstand Bonferroni correction (p_B_ = 0.3) and the association may thus also be a consequence of the many tests. A SNP in BST2 was significant after Bonferroni correction (p_B =_ 0.03). No SNPs in TREX1 were significant.

## Materials and Methods

The sampling of DNA was approved by the Central Denmark Committee on Biomedical Research Ethics, and blood samples were obtained after both written and oral informed consent. The procedure, including the written forms, was also approved by the Science-Ethical committee. Records of all written approvals are kept at the clinical departments. For the present analyses, we employed a previously described cohort of 350 verified MS cases and 500 controls from Western Denmark [14]. The analyses involved were genetic epidemiology, where SNPs were typed on a mass-spectrometry-based Sequenom platform (San Diego, CA) and statistically analyzed for association with disease [14]. The SNPs were selected as having fairly frequent less common alleles. Three plexes were used for the analyses, involving a total of 52 critically evaluated SNP assays. For most of the genes (*TRIM5, TRIM22, APOBEC3*) it was apparent that the group of heterozygotic persons had a relatively low frequency of MS. We therefore pooled them with the homozygotes with few MS cases. For *BST2* it seemed that heterozygotes had an intermediate frequency of disease. Therefore, we analyzed allele frequencies instead. Χ^2^-tests were used in the statistical analyses. P-values were Bonferroni corrected for the multiplicity of testing.

## Results

**Figure 1:**
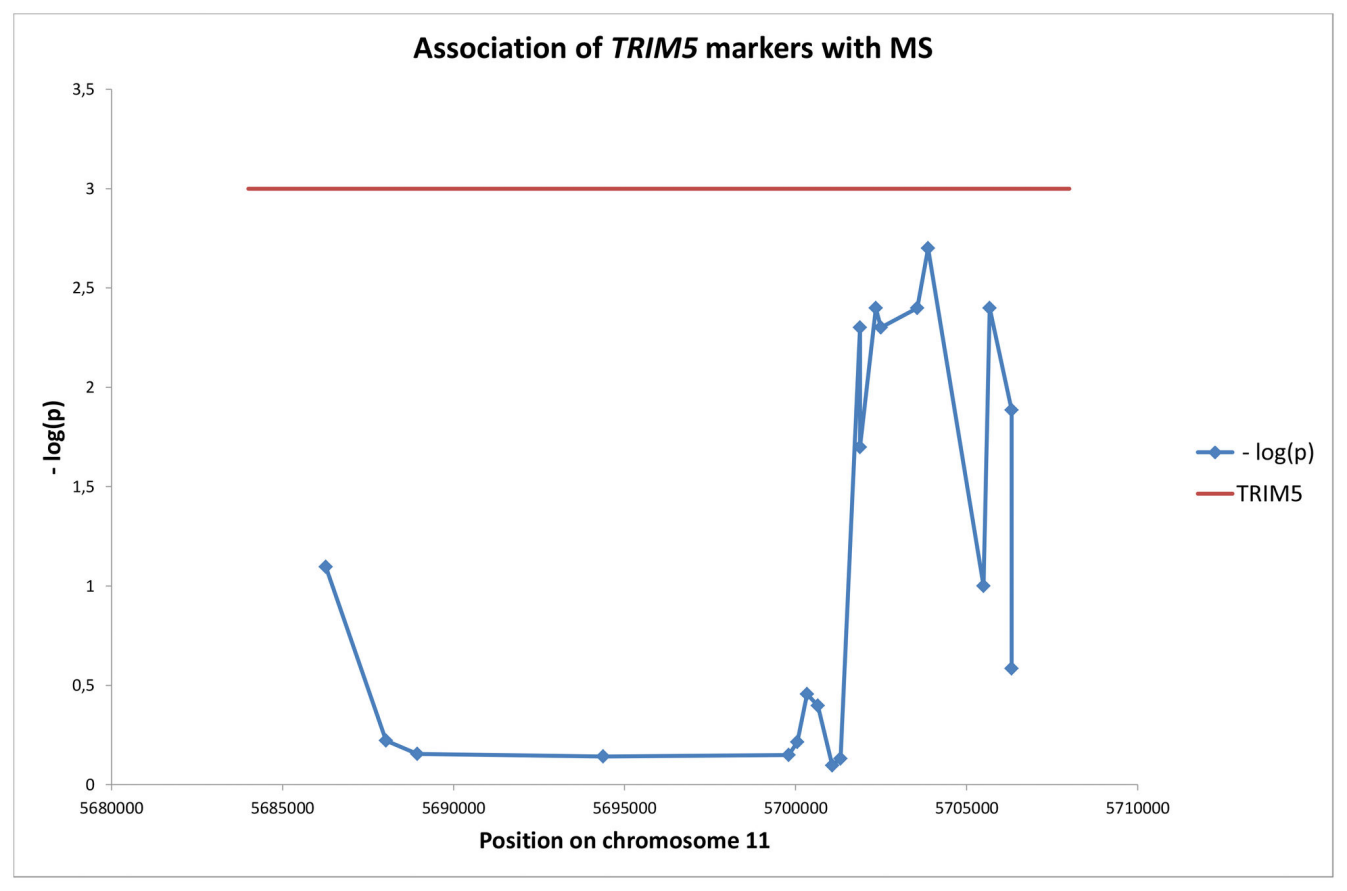
Location of markers in TRIM5 and their association with MS. Data from Table 1.

Initially, we investigated the association of additional markers in *TRIM5* on chromosome 11 with MS. The results are shown in Table 1. It is clear that several markers in this gene were associated with disease. For rs12287199/TRIM5 the association was sufficiently strong to allow for Bonferroni correction for the 20 *TRIM5* tests performed as shown in Table 1 (p_B_ = 0.04). Figure 1 shows the location of the SNPs on chromosome 11 and the negative logarithm of the p-value for their association with the disease. The significant SNPs were located in the rightmost (5’) part of the gene, near the RING finger domain. Presumably, the significant region constitutes one haplotype block, and the resolution within such a block is limited.

**Table 1:** Association of markers in the *TRIM5* gene region with MS.

SNP	R/P^1^	Position^2^	OR (95%CI)^3^	p-value^4^
rs28381981	C/T	5686266	0.70 (0.46-104)	0.08
rs11820502	C/T	5688024	0.93 (0.71-1.22)	0.60
rs11038628	T/C	5688940	0.92 (0.59-1.43)	0.70
rs7116587	C/G	5694377	0.92 (0.59-1.44)	0.72
rs3740994	A/C	5699801	0.86 (0.37-1.97)	0.71
rs28381980	T/A	5700050	0.65 (0.13-3.27)	0.61
rs3740995	C/T	5700340	0.66 )0.27-1.59)	0.35
rs10769175	A/G	5700649	0.88 (0.66-1.18)	0.40
rs11601507	A/C	5701074	0.95 (0.64-1.41)	0.80
rs3740996	G/A	5701317	0.85 (0.31-2.29)	0.74
rs7117107	T/C	5701880	0.67 (0.50-0.89)	0.005
rs17305868	A/G	5701883	0.70 (0.52-0.94)	0.02
rs2880574	G/C	5702343	0.69 (0.50-0.95)	0.004
rs4992801	T/C	5702489	0.66 (0.49-0.88)	0.005
rs12278842	C/G	5703558	0.66 (0.49-0.88)	0.004
rs12287199	T/A	5703870	0.63 (0.47-0.85)	0.002
rs937446	G/A	5705490	0.72 (0.48-1.06)	0.10
rs2133256	G/A	5705675	0.65 (0.49-0.88)	0.004
rs3802981	C/T	5706312	0.68 (0.51-0.92)	0.013
rs3802980	A/G	5706312	0.82 (0.58-1.16)	0.26

1 Restrictive/Permissive alleles.

2 Position on chromosome 11 (NCBI 37.3).

3 Odds ratio and 95 percent confidence interval for the restrictive homozygote and the heterozygote vs. the permissive homozygote.

4 χ^2^-test of the restrictive homozygote and the heterozygote vs. the permissive homozygote. This test is justified by the fact that the heterozygote appears restrictive.

Next, we searched for association between MS and 5 SNPs in the *TRIM22* gene, also on chromosome 11. We found an association between rs7935564/TRIM22 and the disease (Table 2). Again, the association was strong enough to withstand Bonferroni correction (p_B_ = 0.05).

**Table 2:** Association of markers in the *TRIM22* gene region with MS.

SNP	R/P^1^	Position^2^	OR (95% CI)^3^	p-value^4^
rs1498553	C/T	5709028	0.68 (0.50-0.92)	0.014
rs7129909	G/A	5711177	0.78 (0.59-1.02)	0.070
rs7935564	G/A	5718517	0.70 (0.53-0.92)	0.012
rs12282048	G/A	5728212	0.88 (0.64-1.56)	0.43

1 Restrictive/Permissive alleles.

2 Position on chromosome 11 (NCBI 37.3).

3 Odds ratio and 95 percent confidence interval for the restrictive homozygote and the heterozygote vs. the permissive homozygote.

4 χ^2^-test of the restrictive homozygote and the heterozygote vs. the permissive homozygote. This is justified by the fact that the heterozygote appears restrictive.

After this, we turned to the *APOBEC3* genes. These genes lie in a cluster on chromosome 22. 18 SNPs in the region were analyzed (Table 3). We found a positive signal from rs2019907/APOBEC3, located between *APOBEC3B* and *APOBEC3C*. However, after Bonferroni correction, the results were not significant (P_*B*_ = 0.31), indicating that this association could be a results of the multiplicity of testing.

**Table 3:** Association of markers in the *APOBEC3* region with MS.

SNP	R/S^1^	Gene	Position^2^	OR (95%CI)^3^	p-value^4^
rs5750717	A/G	APOBEC3A	39355717	0.81 (0.55-1.20)	0.29
rs6001349	G/T	APOBEC3A_B	39374672	0.89 (0.63-1.24)	0.48
rs2072866	C/G	APOBEC3B	39385809	0.78 (0.59-1.02)	0.066
rs2019907	A/G	APOBECB_C	39389420	0.66 (0.47-0.93)	0.017
rs2142833	G/A	APOBEC3B_C	39392296	0.81 (0.58-1.13)	0.21
rs6001363	C/T	APOBEC3B_C	39392480	0.81 (0.58-1.12)	0.19
rs9607600	C/T	APOBEC3B_C	39395418	0.71 (0.51-1.00)	0.052
rs9611070	G/T	APOBEC3B_C	39395707	0.72 (0.51-1.00)	0.052
rs4315626	C/T	APOBEC3B_C	39399738	0.76 (0.54-1.06))	0.11
rs6001376	C/T	APOBEC3B_C	39407399	0.76 (0.58-1.01)	0.60
rs3884935	G/A	APOBEC3_D	39420093	0.85 (0.56-1.29)	0.45
rs5750728	C/T	APOBEC3F	39440149	0.96 (0.70-1.33)	0.81
rs4821862	C/T	APOBEC3F	39441203	0.93 (0.69-1.26)	0.065
rs6519165	G/A	APOBEC3F_G	39471914	0.77 (0.59-1.01)	0.061
rs5757465	C/T	APOBEC3G	39477123	0.82 (0.56-1.18)	0.28
rs8177832	A/G	APOBEC3G	39477566	0.78 (0.39-1.53)	0.47
rs2413570	C/T	APOBEC3G	39481187	1.00 (0.73-1.36)	0.99
rs2413570	T/C	APOBEC3G	39481187	0.96 (0.71-1.30)	0.79

1 Restrictive/Permissive alleles

2 Position on chromosome 22 (NCBI 37.3).

3 Odds ratio and 95 percent confidence interval for the restrictive homozygote and the heterozygote vs. the permissive homozygote.

4 χ^2^-test of the restrictive homozygote and the heterozygote vs. the permissive homozygote. This test is justified by the fact that the heterozygote appears restrictive.

In testing of 5 SNPS in *BST2* on chromosome 19, rs12979773/BST2 gave the lowest p–value of 0.005, which was significant after Bonferroni correction (p_B_ = 0.03) (Table 4). Testing of rs11797, rs121908117, rs2242150, rs2242158 and rs3135941 in *TREX1* were not significant (results not shown).

**Table 4:** Association of markers in and around the *BST2* with MS.

SNP	R/S^1^	Position^2^	OR (95%CI)^3^	p-value^4^
rs114213263	C/A	17509506	0.72 (0.57-0.90)	0.033
rs13485	C/G	17513926	0.72 (0.55-0.94)	0.011
rs919265	G/C	17514440	0.71 (0.35-1.43)	0.33
rs12979773	T/C	17516764	0.72 (0.57-0.90)	0.005
rs2278233	A/G	17517174	0.78 (0.60-1.01)	0.061

1 Restrictive/Permissive alleles

2 Position on chromosome 19, (NCBI 37.3)

3 Oddsratio of restrictive vs permissive allele

4 χ^2^-test for the restrictive vs the permissive allele.

## Discussion

Here we have tested for the involvement of several retroviral restriction factors in the risk of MS. Our results substantiate the role of *TRIM* genes in MS [14]. SNPs in both *TRIM5* and *TRIM22* were associated with disease, and the associations were strong enough to withstand correction for the multiplicity of testing. The only known triggers of TRIM action are viral particles. Thus, the results indirectly support the hypothesis of a role of virions in MS. As the TRIM5 protein exclusively recognizes incoming particles, the findings also suggest that particle entry is of importance for the viral role in MS. TRIM22 protein also seems to influence formation and budding of virions. *TRIM5* and presumably *TRIM22* play an active role in inducing the innate immune system [17].

We have not been able to demonstrate a statistical interaction of the *TRIM*s with viral loci previously demonstrated to be involved in MS [14,18,19]. Rather the effects appear to be largely additive or non-existing (results not shown). Thus, we can only assume these loci are involved.

We also provided evidence that *BST2* influence the risk of MS. The product of this gene, Tetherin, keeps the location of sensitive retroviral particles closely cell-associated, and thus prevents cell-free spreading. The results point to moderate effects of this factor (all ORs > 0.7). This could reflect that the natural variations in *BST2* only have minor influence on its efficiency. Alternatively, Tetherin is only of moderate importance for MS.

Mutations on *TREX1* have been shown to be of importance for the autoimmune diseases Aicardi-Goutieres Syndrome and chilblain lupus [13]. However, we were not able to demonstrate an importance of this gene in MS. Results with the *APOBEC3* genes also at best pointed to a minor role for these genes.

It is interesting that the three restriction genes that seem to be involved in MS all affect particle release and entry. The two genes, for which we could not demonstrate an effect, affect the integrity of the viral nucleic acid. This could point towards a role of particle transfer to bring about disease, while the infectivity of the virions presumably is only of minor importance. This conclusion may seem at odds with the recent observation that HAART (highly active antiretroviral therapy) may interfere with MS [20,21]. However, the results can be reconciled, if the viruses in question are defective very late in the viral entry, for instance in the integration of the proviral DNA in the cell genome.

Neither the *TRIM*s nor *BST2* were mentioned in a recent meta-analysis of genome-wide association analyses [22]. While the discrepancy with our observations seems surprising, the difference probably lies in the cohorts. Our study was performed on a medium-sized, ethnically highly homogenous cohort, while the GWASes were performed on very large, ethnically in-homogenous cohorts. For this reason, our analysis has less statistical power than the GWASes have. On the other hand, because of the population homogeneity, bias is presumably a minor problem in our study, while it could be major in the GWASes. Importantly, the larger size of cohorts in GWASes does not reduce bias. Also, the large number of comparisons made in GWASes necessitates large compensations for the multiplicity of testing. Time will resolve this issue.

We have also investigated the role of restriction genes for retroviruses in rheumatoid arthritis, another disease, whose etiology could involve retroviruses. So far we have not been able to demonstrate an effect of the genes on this disease (B. Hansen et al., unpublished). We are currently investigating the role of these genes for systemic lupus erythematosus.

In conclusion, we have presented genetic data supporting a role of the retroviral restriction genes*, TRIM5, TRIM22* and *BST2* in the autoimmune disease multiple sclerosis. This lends credit to the hypothesis that retroviruses, presumably endogenous, play a role in this disease. Moreover, it is remarkable that at least two of the viral restriction factors, we have found active in MS, also are viral triggers of the innate immune system. Possibly, they contribute to the autoimmune derangement.
