# Correction: Restriction Genes for Retroviruses Influence the Risk of Multiple Sclerosis

**DOI:** 10.1371/annotation/81edbe21-6549-474d-b456-540e0662c6b8

**Published:** 2013-10-24

**Authors:** Bjørn A. Nexø, Bettina Hansen, Kari K. Nissen, Lisa Gundestrup, Thorkild Terkelsen, Palle Villesen, Shervin Bahrami, Thor Petersen, Finn S. Pedersen, Magdalena J. Laska

This article has been republished to remove supporting information files that should not have been published. Please review this article again without supporting information files.

